# Metagenomic Profiling of Fecal-Derived Bacterial Membrane Vesicles in Crohn’s Disease Patients

**DOI:** 10.3390/cells10102795

**Published:** 2021-10-19

**Authors:** Nader Kameli, Heike E. F. Becker, Tessa Welbers, Daisy M. A. E. Jonkers, John Penders, Paul Savelkoul, Frank R. Stassen

**Affiliations:** 1 Department of Medical Microbiology, School of Nutrition and Translational Research in Metabolism (NUTRIM), Maastricht University Medical Center+, 6229 ER Maastricht, The Netherlands; h.becker@maastrichtuniversity.nl (H.E.F.B.); tessa.welbers@mumc.nl (T.W.); j.penders@maastrichtuniversity.nl (J.P.); paul.savelkoul@mumc.nl (P.S.); 2 Department of Medical Microbiology, Faculty of Applied Medical Sciences, Jazan University, Jazan 45142, Saudi Arabia; 3 Department of Internal Medicine, Division of Gastroenterology/Hepatology, NUTRIM school of Nutrition and Translational Research in Metabolism, Maastricht University Medical Center+, 6200 MD Maastricht, The Netherlands; d.jonkers@maastrichtuniversity.nl; 4 Department of Medical Microbiology, Caphri School for Public Health and Primary Care, Maastricht University Medical Centre+, 6229 ER Maastricht, The Netherlands; 5 Department of Medical Microbiology and Infection Control, VU University Medical Center, 1081 HV Amsterdam, The Netherlands

**Keywords:** gut microbiota, bacterial membrane vesicles, Crohn’s disease, metagenomics

## Abstract

Background: In the past, many studies suggested a crucial role for dysbiosis of the gut microbiota in the etiology of Crohn’s disease (CD). However, despite being important players in host–bacteria interaction, the role of bacterial membrane vesicles (MV) has been largely overlooked in the pathogenesis of CD. In this study, we addressed the composition of the bacterial and MV composition in fecal samples of CD patients and compared this to the composition in healthy individuals. Methods: Fecal samples from six healthy subjects (HC) in addition to twelve CD patients (six active, six remission) were analyzed in this study. Fecal bacterial membrane vesicles (fMVs) were isolated by a combination of ultrafiltration and size exclusion chromatography. DNA was obtained from the fMV fraction, the pellet of dissolved feces as bacterial DNA (bDNA), or directly from feces as fecal DNA (fDNA). The fMVs were characterized by nanoparticle tracking analysis and cryo-electron microscopy. Amplicon sequencing of 16s rRNA V4 hypervariable gene regions was conducted to assess microbial composition of all fractions. Results: Beta-diversity analysis showed that the microbial community structure of the fMVs was significantly different from the microbial profiles of the fDNA and bDNA. However, no differences were observed in microbial composition between fDNA and bDNA. The microbial richness of fMVs was significantly decreased in CD patients compared to HC, and even lower in active patients. Profiling of fDNA and bDNA demonstrated that *Firmicutes* was the most dominant phylum in these fractions, while in fMVs *Bacteroidetes* was dominant. In fMV, several families and genera belonging to *Firmicutes* and *Proteobacteria* were significantly altered in CD patients when compared to HC. Conclusion: The microbial alterations of MVs in CD patients particularly in *Firmicutes* and *Proteobacteria* suggest a possible role of MVs in host-microbe symbiosis and induction or progression of inflammation in CD pathogenesis. Yet, the exact role for these fMV in the pathogenesis of the disease needs to be elucidated in future studies.

## 1. Introduction

Inflammatory bowel disease (IBD) comprises Crohn’s disease (CD) and ulcerative colitis. Although the etiology of IBD is unresolved, a three-compartment pathophysiological circuit consisting of 1. the gut microbiota, 2. the intestinal barrier and 3. the intestinal immune system has been suggested to play a critical role [1,2,3,4]. More recently, bacterial membrane vesicles (MVs) have gained attention as a potentially important new player in understanding the intersection of the gut-microbial communities and human health. Gut microbiota can secrete different types of MVs, including outer membrane vesicles (OMVs) and outer-inner membrane vesicles for Gram-negative bacteria, and membrane vesicles for Gram-positive bacteria. MVs can contain lipids, outer membrane proteins, nucleic acids, ATPs, and cytoplasmic and inner membranes. As a result, they play a crucial role in bacterial survival, nutrient sensing, carrying virulence factors, modulation of host immune function, and killing competing bacteria [5,6,7,8,9,10].

Recent studies have strongly indicated a coherence between gut microbiota dysbiosis and CD [11,12,13]. Those perturbations are characterized by a decrease in the diversity of commensal bacteria, for instance with major alterations in members of the phyla *Firmicutes* and *Bacteroidetes* [14,15]. Microbial shifts have also been observed in active disease versus remission within CD patients. For instance, during an exacerbation, severe reductions in *Faecalibacterium prausnitzii* and *Clostridium coccoides* were seen while the prevalence of *Enterobacteriaceae*, and *Bacteroides* spp. were increased [15,16,17]. Studies also suggested that dysbiosis in CD is correlated to a reduction of butyrate-producing bacteria [18,19]. This results in decreased butyrate levels, in turn causing reduced expression of epithelial tight junction proteins and therefore increased epithelial permeability. This might describe one potential mechanism of the role of dysbiosis [15,16]. MVs isolated from different strains of *Bifidobacterium* and *Lactobacillus* may mediate the probiotic impacts of the MV-releasing microbes [20]. Studies investigating the immunoregulatory roles of MVs have unraveled pro- and anti-inflammatory effects [21,22,23].

Despite the suggested importance of the microbiota in the pathogenesis of CD, the role of microbiota-derived MVs in the etiology has been neglected. To investigate a potential role of microbiota-derived MVs in CD pathogenesis, we profiled the origin of MVs isolated from CD patients (both with active disease and in remission) and healthy controls using 16 S ribosomal DNA (rDNA) amplicon sequencing, and compared them to the fecal microbiota composition. 

## 2. Materials and Method

### 2.1. Study Population

From twelve patients with CD (either with active disease n = 6 (Ac-CD), or in remission, n = 6 (Re-CD)) of the IBD South Limburg (IBD-SL) biobank project, fecal samples were collected as described previously [24]. Also, samples from six healthy volunteers (HC) of the Maastricht IBS Cohort (MIBS) were included [24]. CD was diagnosed based on clinical and endoscopic or radiological findings conforming to the ECCO guidelines [25]. Fecal samples were collected by the patients at home, stored at 4 °C, and brought to the hospital within 2 h after defecation. The samples were aliquoted and frozen directly at −80 °C for further analyses. Disease activity was defined by the simple endoscopic score of CD (SES-CD) [26]. Active disease was defined by SES-CD score ≥3, while remission was defined by SES-CD score <3.

### 2.2. Ethical Statement

The patients gave written informed consent prior to participation. The study was approved by the Medical Ethics Committee of Maastricht University Medical Center+, and was executed according to the revised declaration of Helsinki (59th general assembly of WMA, Seoul, Korea, October 2008). The study was registered in the Central Committee on Research Involving Human Subjects (CCMO) registry, under file number NL31636.068.10 (IBD-SL) and NL24160.068.08 (MIBS).

### 2.3. Vesicle Isolation from Fecal Samples

The steps for optimizing a protocol for isolating heterogeneous MV populations from fecal samples (fMVs) were based on previous research performed by Benedikter et al. [27], in which a protocol was designed to isolate MVs from cell culture media and modified by our group for feces MVs [28]. Briefly, 0.5 g of fecal matter was weighed and dissolved in 10 mL filtered phosphate buffered saline (PBS). A centrifugation step was applied (15 min, 4668 rcf, 4 °C) to remove solid debris and cells. After centrifugation, the debris pellet containing bacterial cells was resolved in lysis buffer (ASL buffer, QIAamp DNA Stool Kit 51504, QIAGEN, Hilden, Germany) for further bacterial DNA (bDNA) isolation while the supernatant containing vesicles was applied on ultracentrifugation (100,000× *g* for 2.5 h, at 4 °C) to remove extracellular DNA not associated with fMVs [7], the pellet were re-suspended in 5ml PBS and sequentially filtered, first by 0.45 μm (Acrodisc syringe filters, Pall Life Sciences, New York, NY, USA) and then by 0.2 μm (Minisart© NML syringe filter, Sartorius Stedim Biotech, Gottingen, Germany). To separate vesicles from free molecules, the filtrate was then loaded onto a filter with a molecular weight cut-off of 100 kDa (Amicon Ultra 15 mL Centrifugal Filter Unit, Merck Millipore, Billerica, MA, USA) and concentrated to 250 μL by centrifugation (45 min., 4668 rcf, 4 °C). The filter membrane was additionally rinsed with 250 μL sterile PBS in order to achieve complete MV recovery, and an end volume of 500 µL was used for the next step. 

The next step involved purification of the concentrate by separating the vesicles from free protein. This was achieved by size exclusion chromatography (SEC) with 10 mL sepharose (CL2B) columns (GE Healthcare, Eindhoven, The Netherlands). The concentrated supernatant was loaded onto the column and fractions of 0.5 mL were immediately collected in Eppendorf tubes. In total, 24 fractions of 0.5 mL were collected per sample. Fractions 7–11, containing membrane vesicles [27], were pooled, and particle concentration was determined using nanoparticle tracking analysis (NTA; ZetaView PMX120). Pooled fractions were stored at −80 °C until further analysis. Internal and external MVs-DNA were obtained from pooled fractions by heating extraction at 95 °C for 7 min [29]. 

### 2.4. Visualizing MVs by Cryo-Transmission Electron MICROSCOPY (Cryo-TEM)

Three microliters of isolated vesicles were applied to a glow-discharged holey carbon grid before blotting against filter paper to leave only a thin film spanning the grid holes. The sample was kept at 95% humidity before plunge-freezing in liquid ethane using a Vitrobot (FEI, Eindhoven, The Netherlands). The vitreous sample films were transferred to a Tecnai Arctica Cryo-Transmission electron microscope (ThermoFisher, Eindhoven, The Netherlands). The images were taken at 200 kV with a Falcon camera (ThermoFisher, The Netherlands).

### 2.5. Fecal and Bacterial DNA Isolation

DNA was isolated directly from frozen fecal samples, representing total fecal DNA (fDNA), and from the 4668 rcf pellet after dissolving fecal matter with PBS which represent bacterial cells’ DNA (bDNA). DNA was isolated by repeated-bead-beating (RBB) combined with chemical lysis and a column-based purification method using the QIAamp^®^ DNA Mini kit (Qiagen, Cat. No. 51306) [30]. For bDNA, the obtained pellet from the 5000 rpm was homogenized with 2.0 mL pre-heated (65 °C) ASL buffer of the kit by vortexing. 1.0 mL of this suspension was then added in a 2 mL tube containing 0.5 g of sterile zirconia beads (Ø 0.1 mm, BioSpec, Cat. No. 11079101z), while for fDNA approximately 150 mg of feces was directly added to the beads without this pre-processing step. The DNA isolation procedures were performed according to the manufacturer’s instructions of the kit. The DNA concentration was then measured using the Qubit 3 fluorometer (ThermoFisher Scientific).

### 2.6. Next Generation Sequencing by Illumina MiSeq

Negative controls were included for each isolation batch (PBS for MVs isolation, and PCR-grade water for fecal DNA isolation). A previously published protocol was used for generating amplicon libraries and sequencing [31]. Briefly, the 16S rRNA gene V4 variable region was amplified using 10 pmol of both primers (5′-GTGCCAGCMGCCGCGGTAA*-3′[515 F] and barcoded 5′-GGACTACHVGGGTWTCTAAT*-3′ [806 R]), 5 μL Accuprime buffer II, 0.2 μL Accuprime Hifi polymerase (Thermo Fisher Scientific, Waltham, WA, USA), H_2_O(WMB) and 2 μL DNA in a total reaction volume of 50 µL. The PCR program consisted of an initial denaturation and enzyme activation step at 94 °C for 3 min, then PCR amplification was carried out for 35 cycles for low yield DNA <5 ng, and 24 cycles for high yield DNA >5 ng; amplification cycles ran for 30 s at 94 °C, 45 s at 50 °C and 1 min at 72 °C, finally the program ended with a post-PCR step at 72 °C for 10 min for completion of synthesis of PCR products. PCR amplicon libraries were checked for quality and adequate size on a 1% agarose gel. 

Amplicons were subsequently purified automatically on a Zephyr^®^ G3 NGS Workstation (PerkinElmer, Waltham, MA, USA) using Agencourt AMPure beads (Brea, CA, USA), subsequently quantified by Quant-iT PicoGreen dsDNA reagent kit (Invitrogen, New York, NY, USA), and measured on a Victor3 1424 multilabel counter (PerkinElmer). Amplicons were mixed in equimolar concentrations to a final concentration of 1 ng/µL of pooled DNA to ensure equal representation of each sample, then sequenced on an Illumina MiSeq instrument using the V3 reagent kit (2 × 250 cycles). All V4 16S rDNA bacterial sequences generated in this study were submitted to NCBI databases with accession (PRJNA720101). 

### 2.7. Sequencing Analysis

The online Integrated Microbial Next Generation Sequencing (IMNGS) platform was used for data demultiplexing, length and quality filtering, pairing of reads, and clustering of reads into operational taxonomic units (OTUs) at 97% sequence identity using default settings (www.imngs.org accessed on 25 July 2020). Sequences were conducted from both the 3′ and 5′ sides, and fragments around 250 bases each were extracted after removal of the primers and technical reads. IMNGS is an UPRASE-based analysis pipeline [32]. Demultiplexing was performed by demultiplexer_v3.pl (unpublished Perl script), while pairing, quality filtering and OTU clustering (97% identity) were performed by USEARCH 8.1 [33]. Chimera filtering was performed by UCHIME (with RDP set 15 as a reference database) [34]. Taxonomic classification was performed by RDP classifier version 2.11 training set 15 [35]. Sequence alignment was performed by MUSCLE, and treeing by FastTree [36,37]. Sequences of negative controls were evaluated and compared to the lowest abundant microbial samples to exclude any possible DNA contaminants and to ensure that the results were not driven by potential contaminant taxa. The highest-sequence reads of the negative controls was less than 9000 reads, while the lowest reads of the samples were more than 59,000. Therefore, the negative controls contained far fewer reads and had a completely different composition and diversity (see supplementary Figure S5).

### 2.8. Richness, Diversity, and Taxonomy

To normalize the data, Rhea package version 1.6 with RStudio (version 1.3.1056) was used [38]. It was also used to detect alpha- and beta-diversity and taxonomical binning. After discarding the negative control reads, normalization of the data was performed based on the lowest sequence reads of the samples, which was 59,527, thereafter OTUs were counted. 

### 2.9. Statistical Analysis

Data analysis was performed with RStudio (version 1.3.1056) and GraphPad Prism (version 5.03). For distance matrix analysis of beta diversity, a permutational multivariate analysis of variance (PERMANOVA) was applied to detect significant differences between the groups. The Mann–Whitney test was performed for differences in bacterial richness and diversity, and for differences in the relative abundance of phyla, families and genera. *p* values are indicated as follows: **** *p* < 0.0001; *** *p* < 0.001; ** *p* < 0.01; and * *p* < 0.05. 

## 3. Results

### 3.1. Study Population

In Table S1, the general characteristics of both CD patients and healthy controls, are shown. No demographic differences were seen between the groups. Most of the CD patients belonged to phenotype B1 with (4/6) and (5/6) for active and remission, respectively (Tables S1 and S2). Importantly, most patients used immunosuppressants, three used antibiotics, and none of the healthy controls used any of these drugs.

### 3.2. MVs Characterizations

The fMVs were characterized based on protein concentration and particle quantification followed by TEM visualization. Quantification of fMVs was done by nanoparticle tracking analysis (NTA). The particle concentration of isolated vesicles was significantly higher in healthy controls compared to CD patients (*p*-value < 0.05). Particle concentrations from healthy volunteers ranged from 3.7 × 10^10^–3.14 × 10^11^ particles/g wet weight of fecal samples with a median of 1.8 × 10^11^, while in Re-CD patients particles ranged from 3.29 × 10^10^–2.2 × 10^11^ with a median of 1.03 × 10^11^, and in Ac-CD from 2.97 × 10^10^–2.6 × 10^11^ with a median of 7.5 × 10^10^. The vesicles were visualized by cryo-TEM (Figure S1). 

### 3.3. Microbial Composition and Diversity

To investigate how the bacterial origin of fMVs relates to the bacterial composition of the intestinal microbiota, DNA was isolated either directly from fecal samples (fDNA), from pelleted bacteria (bDNA) or from isolated MVs (MV-DNA). A total of 7,505,735 sequence reads with a median of 84,392 reads per sample (range from 59,527 to 121,131) were obtained upon amplicon sequencing of the 16S rRNA V4 gene region of the samples. All sequences were subsequently clustered into 361 OTUs based on 97% similarity.

**Figure 1:**
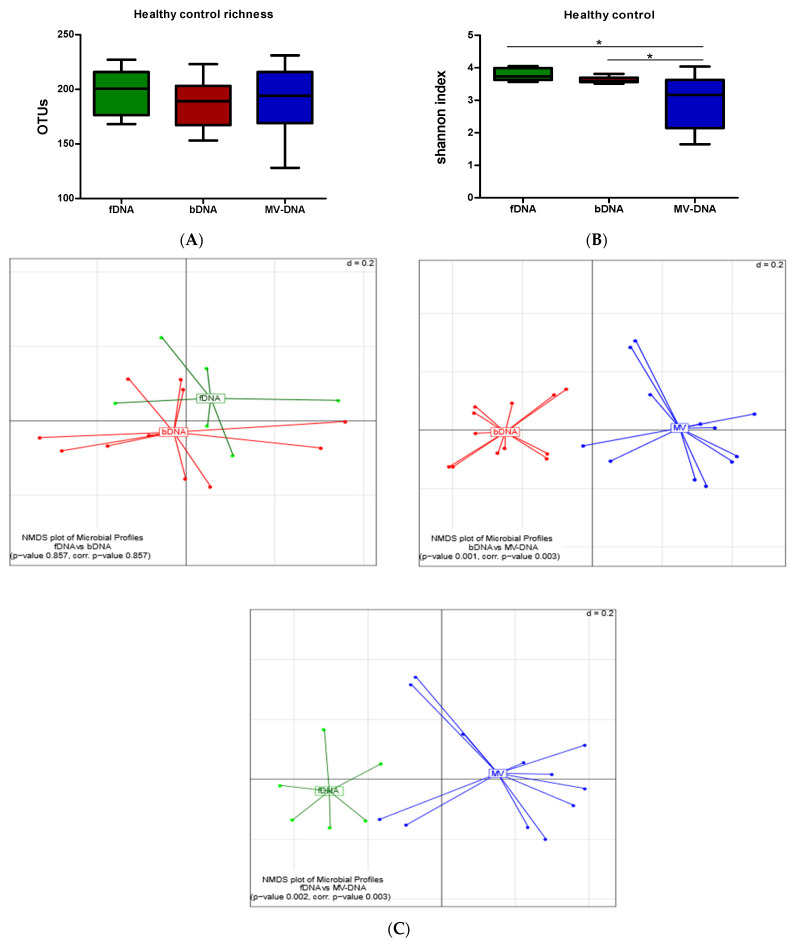
Microbial richness and alpha diversity of DNA obtained from HC to show the differences between the three different conditions: OTU numbers were used to represent the richness of samples. (**A**) Graphs show the richness among the three groups (fDNA, bDNA, and MV-DNA). (**B**) Shannon indices as an indicator for alpha diversity (microbial diversity within the samples). (**C**) Pairwise beta diversity of microbial compositions: non-metric multidimensional scaling (NMDS) calculated from the generalized UniFrac dissimilarity matrix (red: bDNA; green: fDNA; blue: MV-DNA). Results shown as boxplots with whiskers, and *p*-value indicated as * < 0.05.

**Figure 2:**
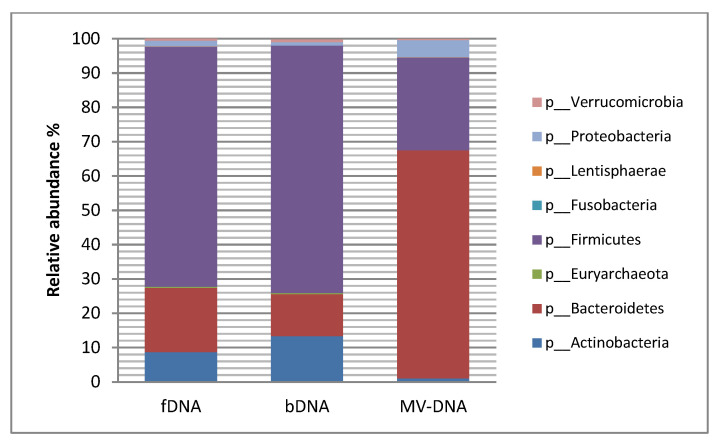
Comparison of relative abundances of the most dominant phyla between the three DNA conditions. The graphs represent the relative differences in healthy controls. (fDNA) DNA obtained directly from fecal samples, (bDNA) DNA obtained from pellet of dissolved feces in PBS–bacteria DNA, and (MV-DNA) DNA obtained from MV fractions.

To evaluate the possible differences in microbial origin between MV-DNA, fDNA and bDNA, we first assessed the compositions within healthy individuals. While no differences were observed in microbial richness between the three sample types (Figure 1A), a significant reduction in microbial diversity (Shannon index) in MV-DNA was observed when compared to the other two sample types (Figure 1B). Also, when the generalized UniFrac was calculated as a measure of inter-sample distance in microbial community structure, MV-DNA was significantly separated from fDNA and bDNA, as visualized by non-metric multidimensional scaling (NDMS). Statistical testing using PERMANOVA indeed confirmed a significant difference in the microbial community structure of MV-DNA samples (adjusted *p*-value = 0.003). No differences were observed in composition between fDNA and bDNA (Figure 1C). Intriguingly, with respect to the microbial composition, fDNA and bDNA were dominated by the Gram-positive phylum *Firmicutes* (mean of relative abundance for fDNA 70%; bDNA 72.1%). In contrast, in MV-DNA the *Bacteroidetes* phylum was dominant, with a mean prevalence of 66 % (Figure 2, Table 1). On the other hand, *Firmicutes* and *Actinobacteria* show significantly less abundance in MV-DNA, which was 3- and 10-fold less, respectively (Figure 2, Figures S2 and S3, Table 1).

**Figure 3:**
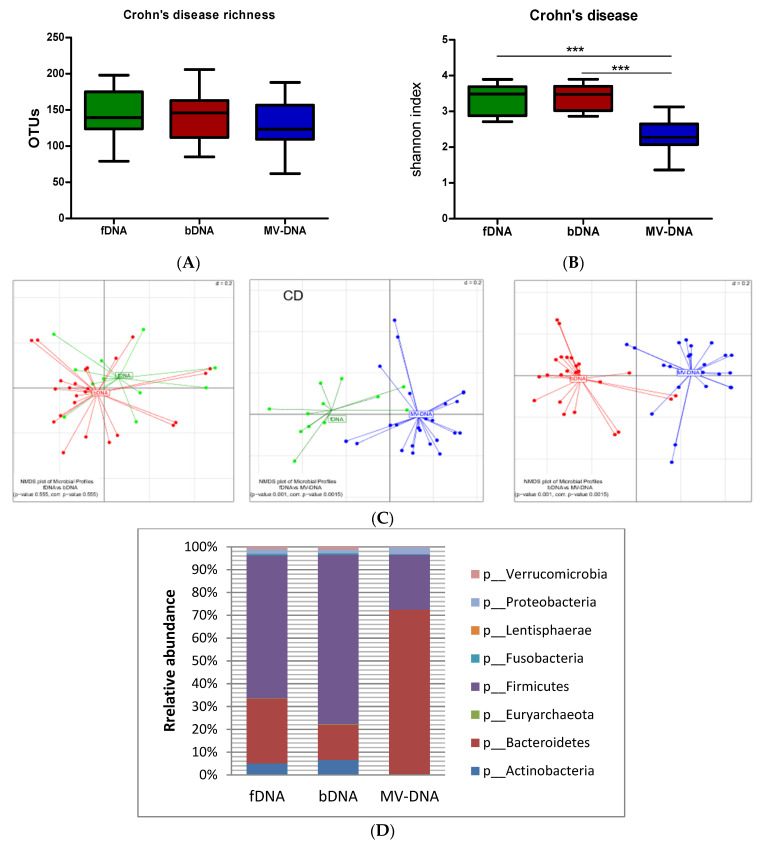
Microbial richness and alpha diversity of DNA obtained from CD to show the differences between the three sample types: OTUs numbers were used to represent the richness of samples. (**A**) Graphs show the richness among the three groups (fDNA, bDNA, and MV-DNA). (**B**) Shannon indices as an indicator for alpha diversity (microbial diversity within the samples). (**C**) Pairwise beta diversity of microbial compositions: non-metric multidimensional scaling (NMDS) calculated from the generalized UniFrac dissimilarity matrix (red: bacterial DNA; green: fecal DNA; blue: vesicles) from the PERMANOVA test used to determine the *p*-value. (**D**) Relative abundances of the most dominant phyla in CD among the three sample types. Results shown as boxplots with whiskers, and *p*-value indicated as (*** < 0.001).

In CD patients, the microbiota compositions of MV were also significantly different compared to fecal and bacterial as seen by beta-diversity analysis (Figure 3). Although microbial richness analysis does not show differences between the three samples types, the microbiota reveals a significantly higher diversity in fDNA and bDNA compared to MV-DNA (Figure 2). Similar to HC, in CD, MV-DNA microbiota is dominated with *Bacteroidetes*, while *Firmicutes* is more abundant in fDNA and bDNA (Figure 3, Figures S2 and S3).

**Figure 4:**
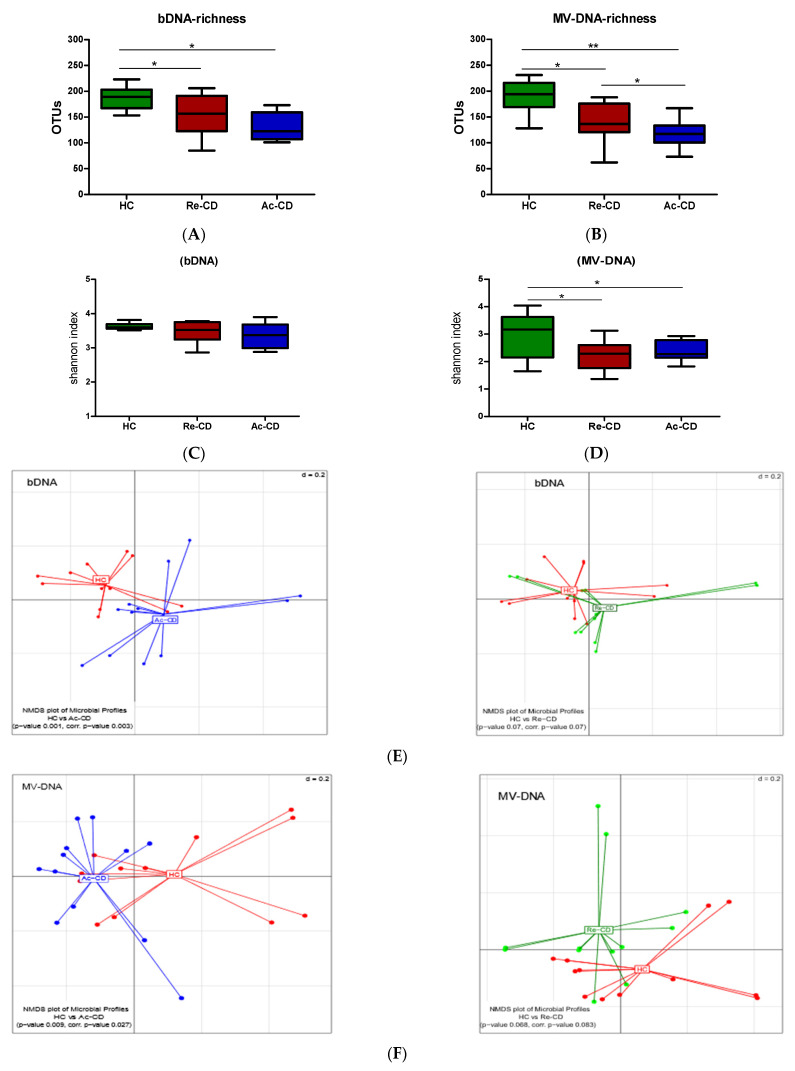
Microbial compositions and diversity of MV-DNA and bDNA of CD patients in comparison to HC (green: HC; red: Re-CD; blue: Ac-CD). (**A**,**B**) Microbial richness in bacterial DNA and MV DNA. (**C**,**D**) Alpha diversity as indicated by Shannon indices. (**E**,**F**) Beta diversity of microbial compositions: non-metric multidimensional scaling (NMDS) calculated from the generalized UniFrac dissimilarity matrix. Pairwise comparison revealed a significant distance between HC and Ac-CD in bacterial DNA (**E**) and vesicles DNA (**F**), the PERMANOVA test used to determine the *p*-value. Results show as boxplots with whiskers. A Mann–Whitney test is used for statistics; *p*-value indicated as (* < 0.05, ** < 0.01).

Thereafter, we analyzed the microbial composition in CD patients as compared to HC. Since the differences between fDNA and bDNA compositions were only marginal, we decided to use only the bDNA readouts to compare it to MV-DNA. The microbial richness was significantly lower in Re-CD as compared to HC, and was even further decreased in Ac-CD in both the MV and bacterial fractions (Figure 4A,B). The Shannon indices, which represent microbial diversity within samples, showed a significant reduction in CD patients in MV-DNA, but not in bDNA (Figure 4C,D). The overall microbial community structure (generalized UniFrac) of samples from HC and Ac-CD was statistically significantly different in both bDNA and MV-DNA fractions (*p*-value of 0.003 and 0.03, respectively; Figure 4E,F). The microbial community structure of samples from re-CD as compared to HC was neither significantly different in the bDNA nor in the MV-DNA factions upon adjustment for multiple comparisons (*p* = 0.07 and 0.083, respectively).

We next examined the composition of bacterial fMVs and bacterial fractions of both CD and HC patients in more detail by testing for differentially abundant genera and families. 

**Figure 5:**
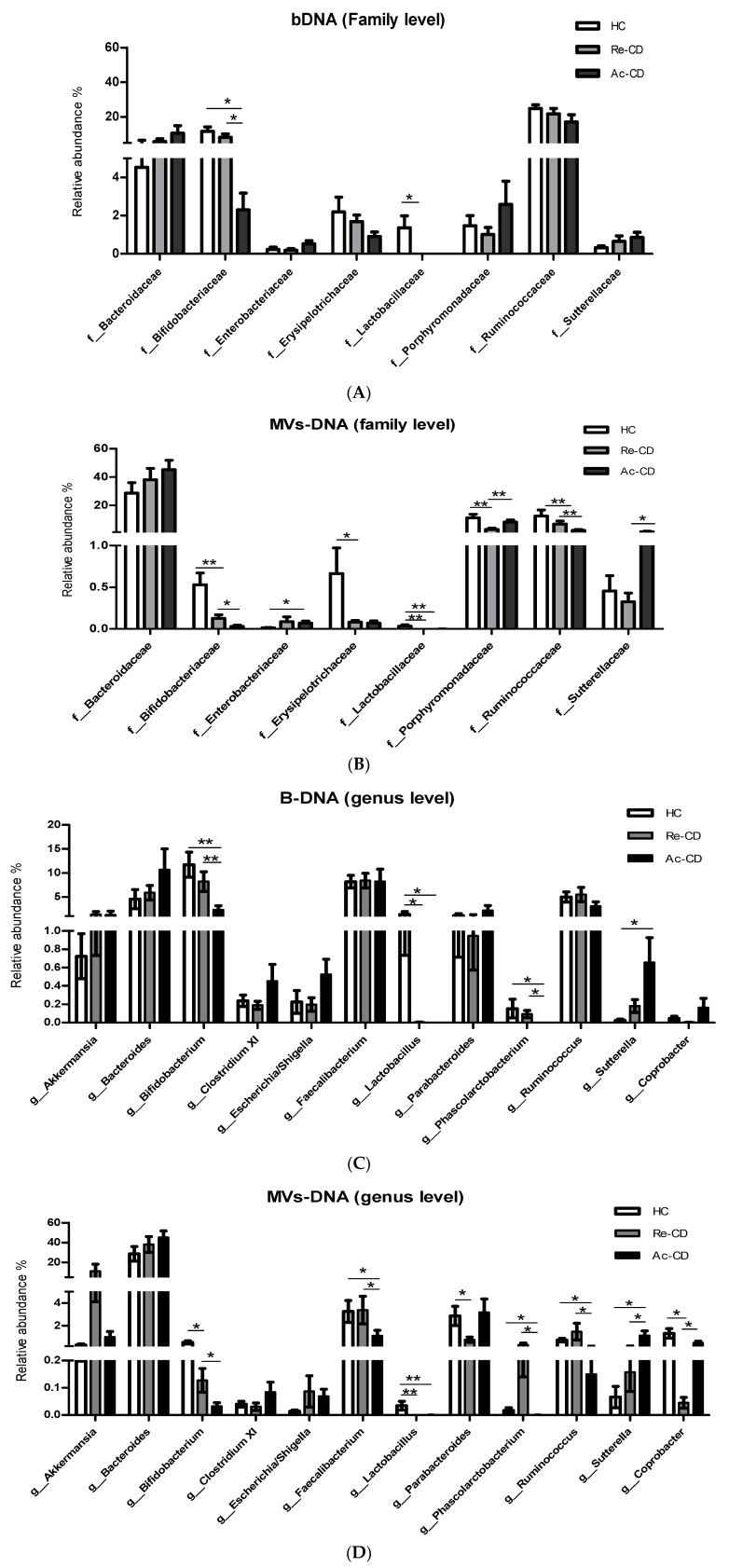
Relative abundance of microbial compositions of MV-DNA and bDNA at the family and genus levels. (**A**,**C**) The relative abundances of the most important families and genera that show clinical importance in IBD, in DNA obtained from bacterial fractions. (**B**,**D**) Relative abundances of microbial compositions of DNA derived from MV fractions. Results represented as means ± SEM. A Mann–Whitney test was used for statistics; *p*-value indicated as (* < 0.05, ** < 0.01).

In CD patients, a significant reduction in the relative abundance in the family *Bifidobacteriaceae* and genus *Bifidobacterium* was observed in both bacterial and MV fractions. This reduction became even more pronounced among patients with active disease (Figure 5A–D). Another striking finding was the almost complete absence of the genus *Lactobacillus* and even the entire family *Lactobacillaceae* in both the bacterial and fMVs fractions of all CD patients as compared to HC.

Although the above-mentioned differences were observed in both bacterial and MV fractions, more profound differences were observed in the MVs. The abundance of *Sutterellaceae* was significantly increased in patients with active disease, and at the genus level a significant trend could also be observed with lowest levels of *Suterella* among HC, intermediate levels in Re-CD and the highest levels among Ac-CD. Significant differences were also observed for *Enterobacteriaceae*, *Porphyromonadaceae, Erysipelotrichaceae, Lactobacillaceae,* and *Ruminococcaceae* at the family level (Figure 5B), whereas at the genus level the reduced abundance of *Faecalibacterium* among ac-CD was most striking.

## 4. Discussion

It is well established that the gut microbiota provide a variety of health-related functions based on symbiotic interactions between the host and the microbiota [39]. Alternatively, currently there is compelling evidence that microbial dysbiosis, which is characterized by alterations in the composition and/or the function of the microbiota, results in a disbalance with the host immune system [14,40]. Consequently, diseases like asthma, allergies, cardiovascular diseases and, in particular, IBD will develop [14,41]. With respect to the latter, many studies have shown that the composition of the microbiota is altered in CD patients compared with that in healthy subjects [14,15,42]. However, convincing evidence showing a direct causal relationship between microbial dysbiosis and CD is lacking, and results show a great diversity in dysbiosis. More recently, it was suggested that not only the bacteria themselves, but also small nanosized vesicles released by almost all bacteria may play a role in health and disease [6,43]. However, the relationship between the composition of the bacterial microbiota and the membrane vesicles released by the bacteria has largely been unexplored. Here, we demonstrate a remarkable difference in the bacterial composition of the gut microbiota and the relative abundance of membrane vesicles within the same microbiota. Also, we explored whether the compositions of both the bacteria and the membrane vesicles was altered in patients with CD.

In the first part of this study, we determined the richness and diversity of three different fecal sample types (whole feces fraction, bacterial fraction and MV fraction) from healthy volunteers. No differences in richness were observed between all three sample types, but diversity was significantly lower in MV-DNA when compared to fDNA and bDNA. Moreover, MV-DNA could be clearly separated from fDNA and bDNA when beta diversity was calculated. Even more intriguing was the difference in the relative abundances of the most dominant phyla between MVs and the bacteria. In both the fDNA and the bDNA samples *Firmicutes* were the most dominant phylum, while *Actinobacteria* could also easily be identified. In the MV-DNA samples, however, *Bacteroidetes* was the dominant phylum, while *Actinobacteria* could hardly be found. Others recently also determined the composition of MVs in different sample types by metagenomic profiling. In several studies, the highest abundance was found for *Firmicutes* in stool [44] and serum samples [45], which is in contrast to our findings. In these studies, they only determined the bacterial origin of the MV, but did not compare this to the actual bacterial composition, as in the current paper. Such a pairwise comparison was made by Yang et al. [46]. Although correlations were found between the presence of MV and bacteria in stool samples, differences were also observed, although they were not in complete alignment with our data. In a similar animal study, Kang et al. reported that the MV present in murine stool samples were almost exclusively proteobacteria-derived, while the relative abundance of bacteria in the sample was more diverse, with *Firmicutes* being the most prevalent phylum. What causes these discrepancies is yet unknown, although it cannot be excluded those differences in MV isolation play a crucial role. Moreover, the processing of fecal samples and DNA extraction methods need to be standardized since it was shown that variations in either processing or extraction may have a large effect on the outcome of metagenomic analyses [47].

In the second part of this study, we determined whether the composition of the gut microbiota was altered in patients with Crohn’s disease. Several studies have demonstrated differences in microbial composition of the gut microbiota among IBD patients in comparison to healthy individuals, describing a global compositional change but also the presence of potential pathogens. For instance, *Faecalibacterium* is often reduced in CD patients, while *Enterobacteriaceae* are increased [48]. Yet, the composition of MV has barely been explored in CD patients. Here we demonstrated that, like in healthy controls, the richness between the three sample types was comparable, while the diversity was significantly lower in the MV fractions. Also, calculation of the beta diversity allowed a clear separation between MV-DNA on one hand and fDNA/bDNA on the other. Likewise, the relative abundance of the most important phyla was clearly different in the MV and fDNA/bDNA samples. Overall, these data demonstrate an unexpected diversity between the MV and bacteria in in term of composition both in healthy controls and in CD patients. 

Several gut microbiota composition studies and their correlations to CD were performed on DNA isolated from fecal samples; however so far these studies could not provide definitive cause-effect mechanistic relationships [49]. Nevertheless, bacterial products are thought to have an impact on causation of IBD and other related diseases. One of the main products are MVs which are suggested to modulate host–microbe interactions [50,51]. Here, we examined whether the composition of the microbiota was different between HC and CD patients (active disease or in remission). In terms of richness, we observed a significant decrease in the bacteria and the MVs in patients in remission when compared to HC, and this was even more pronounced in patients with active disease. A similar trend was observed when beta diversity was calculated. This allowed a significant separation for both MVs and bacteria between AC-CD patients and HC, while the difference between Re-CD and HC was less pronounced. Alpha diversity was also decreased in MV, but not in bacteria. Overall, this confirms the results from other studies also showing a decrease in the richness of the intestinal microbiota in CD patients, which depends on the disease state of the patients [40,52,53,54]. 

We also determined whether changes could be observed at the family and genus levels. Many different studies have revealed enriched or lower fecal concentrations of members of the most important phyla. In particular, changes in the abundances of *Faecalibacterium, Clostridium, Bifidobacterium* and *Lactobacillus* have been reported [11,16]. In our study differences in abundance were noted when the bDNA factions were analyzed at the family and genus level. In Ac-CD, significant reductions in the abundance of *Bifidobacterium*, *Lactobacillus* and *Phascolarctobacterium* were noted. Most of these genera have been shown to protect the host from mucosal inflammation, e.g., by downregulating the release of inflammatory cytokines [55,56,57,58], and lower abundances have been found frequently in IBD patients. Alternatively, *Sutterella* was more abundant in Ac-CD than Re-CD and HC. A recent study has demonstrated an IgA-degrading effect of *Sutterella spp* in ulcerative colitis [59]. Elevated abundance of *Sutterella* was also observed in IBD patients, which is proposed to correlate with IgA deficiency among IBD patients [60,61]. Nonetheless, in contrast to earlier reports we could not find significant differences in the abundance of *Enterobacteriaceae* of *Faecalibacterium* in the bDNA fractions.

We also performed a taxonomic profiling of the MV isolated form fecal samples. In line with the changes observed in the bDNA samples, the abundance of MVs derived from *Bifidobacterium*, *Lactobacillus* and *Phascolarctobacterium* were also significantly reduced in Ac-CD patients. Yet, significant differences were also noting for other genera. For example, a significant decrease in the abundance of *Faecalibacterium prausnitzii* and *Ruminococcus,* which are known as anti-inflammatory organism and acetate producers, respectively [62,63], was exclusively seen in the MV fractions. The abundance of *Erysipelotrichaceae* was markedly reduced in CD patients irrespective of disease status. The *Erysipelotrichaceae* family was shown to cause inflammation by degrading the protective intestinal mucus layer and secreting many enzymes that can worsen inflammation in different ways. As such, it may play a role in inflammation-related diseases like IBD [64]. Indeed, enhanced levels of *Erysipelotrichacea* have been found in mice with TNF-driven Crohn’s disease-like transmural inflammation. However, lower levels were observed in new-onset CD patients [12]. Likewise, significantly lower levels have been observed in patients who experienced recurrence of CD [65]. In the current study, we also found a slight, non-significant decrease in the bDNA samples, but in the MV-DNA samples the levels of *Erysipelotrichaceae* were markedly reduced in both Ac-CD and Re-CD. Additional studies are needed to further elucidate the role of *Erysipelotrichacea* in IBD or other inflammation related-diseases. 

In conclusion, the data presented here provide a novel layer of complexity in the pathophysiology of inflammation-related disorders like CD. We showed a striking difference in the composition of the bacterial microbiota and bacteria-derived MV herein. In particular, the *Firmicutes/Bacteriodetes* ratio was remarkably different between the bacterial/fecal factions and the MV fraction. Moreover, this is the first study which demonstrates that in addition to changes in the intestinal bacterial composition, the MV composition changes in CD patients. Although we realize that the current data were obtained in only a small patient group and need to be confirmed in new prospective longitudinal and mechanistic studies to further unravel the importance of these findings, they provide promising new research targets for the discovery of therapeutic agents and drug delivery systems in the IBD field [43,50].

## Figures and Tables

**Table 1:** Significantly different phyla in MV microbiome compared to fDNA and bDNA compositions in HC. MAV (mean abundance value). A Mann–Whitney test was used to determine the *p*-value.

Phylum	MAV of HC %			*p*-Value of fDNA/bDNA	*p*-Value of fDNA/MV-DNA	*p*-Value of bDNA/MV-DNA
	fDNA	bDNA	MV-DNA			
p__Actinobacteria	8.665	13.35	1	0.3254	0.0009	0.0001
p__Bacteroidetes	18.69	12.22	66.5	0.08	0.004	0.0001
p__Firmicutes	70	72.1	27	0.5	0.001	0.0001
p__Proteobacteria	1.707	1.013	5	0.2	0.9	0.3
p__Euryarchaeota	0.3482	0.3087	0.006617	0.8	0.22	0.04
p__Fusobacteria	0.003017	0.000227	0.001063	0.1	0.1	0.17
p__Lentisphaerae	0.01331	0.000988	0.08437	0.9	0.3	0.1
p__Verrucomicrobia	0.8032	0.7239	0.3012	0.8	0.22	0.1

## Data Availability

The data underlying this article are available in the article and in its online supplementary material.

## Supplementary Materials

The following are available online at https://www.mdpi.com/article/10.3390/cells10102795/s1, Figure S1: cryo-TEM images. (A) vesicles derived from feces of HC. (B) from Ac-CD, and (C) from Re-CD, Figure S2: Comparison of relative abundances of the most dominants phyla. The graphs represent the relative differences between healthy controls and CD patients (remissive and active) based on DNA obtained (A) from feces, (B) from bacteria pellet, and (C) from MVs. Firmicutes and Bacteroidetes which are the most abundant don’t show significant differences, while Actinobactria, Fusobacteria, and Proteobacteria display significant changes especially in MV-DNA. The graphs (D), (E), and (F) demonstrate altering in ratio between bacteria and their corresponding vesicles in HC, Re-CD, and Ac-CD, respectively. Results represent as means ±SEM. Student t-test is used for statistics; p-value indicates as (* < 0.05, ** < 0.01, *** < 0.001), Figure S3: The microbial compositions of DNA at Phyla level. (A) microbial composition of DNA obtained directly from fecal samples. bars 1-6 from healthy individuals, 7-12 from Crohn’s patients in remissive state Re-CD, 13-18 from Crohn’s patients in active state Ac-CD. (B) microbial compositions of DNA obtained from bacterial pellet, each sample were analyzed twice (two different isolation from each individual fecal sample for reproducibility); bars 1-12 represents healthy individuals, 13-24 from Re-CD, and 25-36 from Ac-CD. (C) Microbial composition of DNA isolated from MVs, all samples also analyzed twice, bars 1-12 from HC, 13-24 from Re-CD, and 25-36 from Ac-CD. Firmicutes phylum are more dominant in fecal and bacterial DNA while Bacteroidetes are abundant in MVs-DNA, Figure S4: The relative abundance of microbial compositions at genus level. all samples also ran twice (two different isolation from each individual fecal sample for reproducibility). (A) microbial compositions of DNA obtained from bacterial pellet; each sample were analyzed in twice. Bars 1-12 represents healthy individuals, 13-24 from Re-CD, and 25-36 from Ac-CD. (B) Microbial composition of DNA isolated from MVs; bars 1-12 from HC, 13-24 from Re-CD, and 25-36 from Ac-CD. Each two bars in a row represent one individual, Figure S5: Beta diversity of highest negative controls compare to the lowest sequences reads of samples. It shows that the microbial compositions of negative controls have completely different compositions compare to the samples and the microbial compositions of samples are not driven by potential contaminated DNA. Table S1: Baseline characterizations of the samples: Healthy Control (HC) and Crohn’s Disease (CD) patients (CD), Table S2: Disease characterizations of the Crohn’s Disease (CD) patients.
