# Supplementary material for: Metagenomic Profiling of Fecal-Derived Bacterial Membrane Vesicles in Crohn’s Disease Patients
Source: Cells. 2021 Oct 19;10(10):2795. doi: 10.3390/cells10102795 (PMC8535131; doi:10.3390/cells10102795)
Supplement: Supplementary file 1 [file cells-10-02795-s001.zip › cells-1374588-supplementary.pdf]

Supplementary data:

Table S1: Baseline characterizations of the samples: Healthy Control (HC) and Crohn's Disease (CD) patients (CD)

	CD (12)		HC (6 samples)
	**Active (6 samples)**	**Remission (6 samples)**	
Gender (male %)	50%	16%	16%
Age: median (range)	47.5 (25-63)	49.5 (38-67)	41.5 (25-50)
Smoking: (%)<br>Never (N)<br>Yes (Y)<br>Stop > 6 month (> 6)<br>Stop < 6 month (< 6)	 50% (N)<br>16.7% (Y)<br>16.7% (> 6)<br>16.7% (< 6)	 50% (N)<br>33.3% (Y)<br>16.7% (> 6)	 50% (N)<br>16.7% (Y)<br>33.3% (> 6)

Table S2: Disease characterizations of the Crohn's Disease (CD) patients

	CD (12)	
	**Active (6 samples)**	**Remission (6 samples)**
Age at diagnosis: (%)<br>A1: ≤ 16y A2: 17-40y<br>A3: > 40y	16. 0 (A1). 50.0 (A2)<br>33.3 (A3)	66.7 (A2)<br>33.3 (A3)
Disease Phenotype: (%)<br>B1: non-stricturing/non-penetrating<br>B2: structuring. B3: penetrating	66.7 (B1)<br>16.6 (B2, B3)<br>16.6 (B3)	83.3 (B1)<br>16.7 (B3)
Disease location: (%)<br>L1: (ileal) L3: (ileocolonic). L2: (colonic)	50.0 (L1) 16.7 (L2)<br>33.3 (L3)	33.3 (L1) 16.7 (L2)<br>50.0 (L3)
Medication use: (%)<br>Antibiotics<br>IBD drugs: Biological<br>Other drugs	 33.3<br>83.3<br>16.7	 16.7<br>16.7<br>66.7
SES-CD score: (%)<br>0-2 remission 3-6 mild endoscopic activity	33.3 (mild)<br>50.0 (moderate)<br>16.7 (severe)	 100 remission

7-15 moderate endoscopic activity<br><br>>15 severe endoscopic activity		

(A)

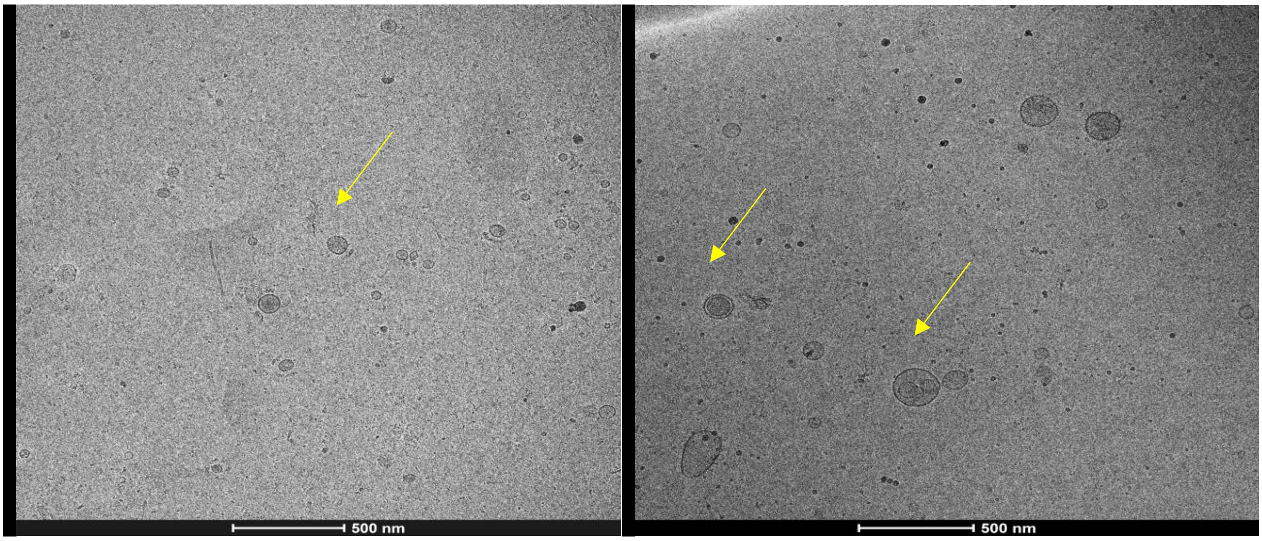

(B)

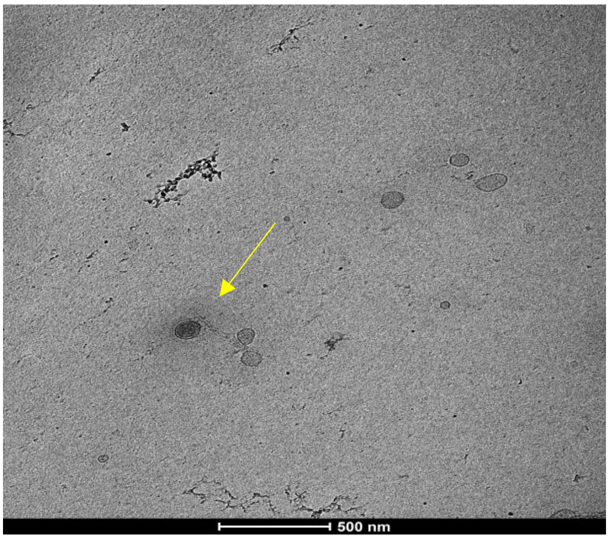

(C)

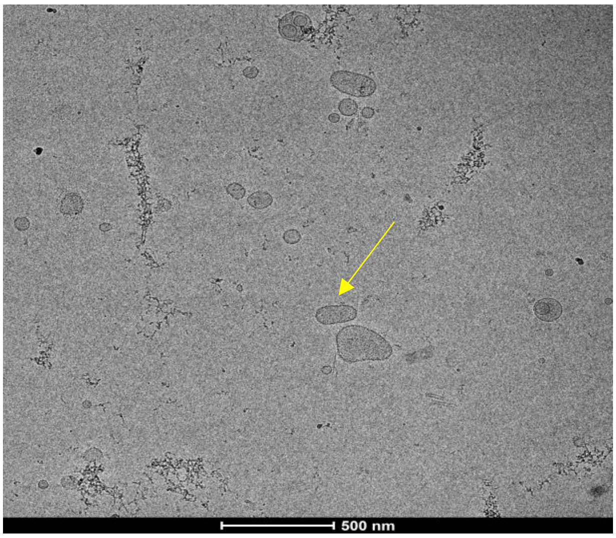

Figure S1: cryo-TEM images. A) vesicles derived from feces of HC. B) from Ac-CD, and C) from Re-CD

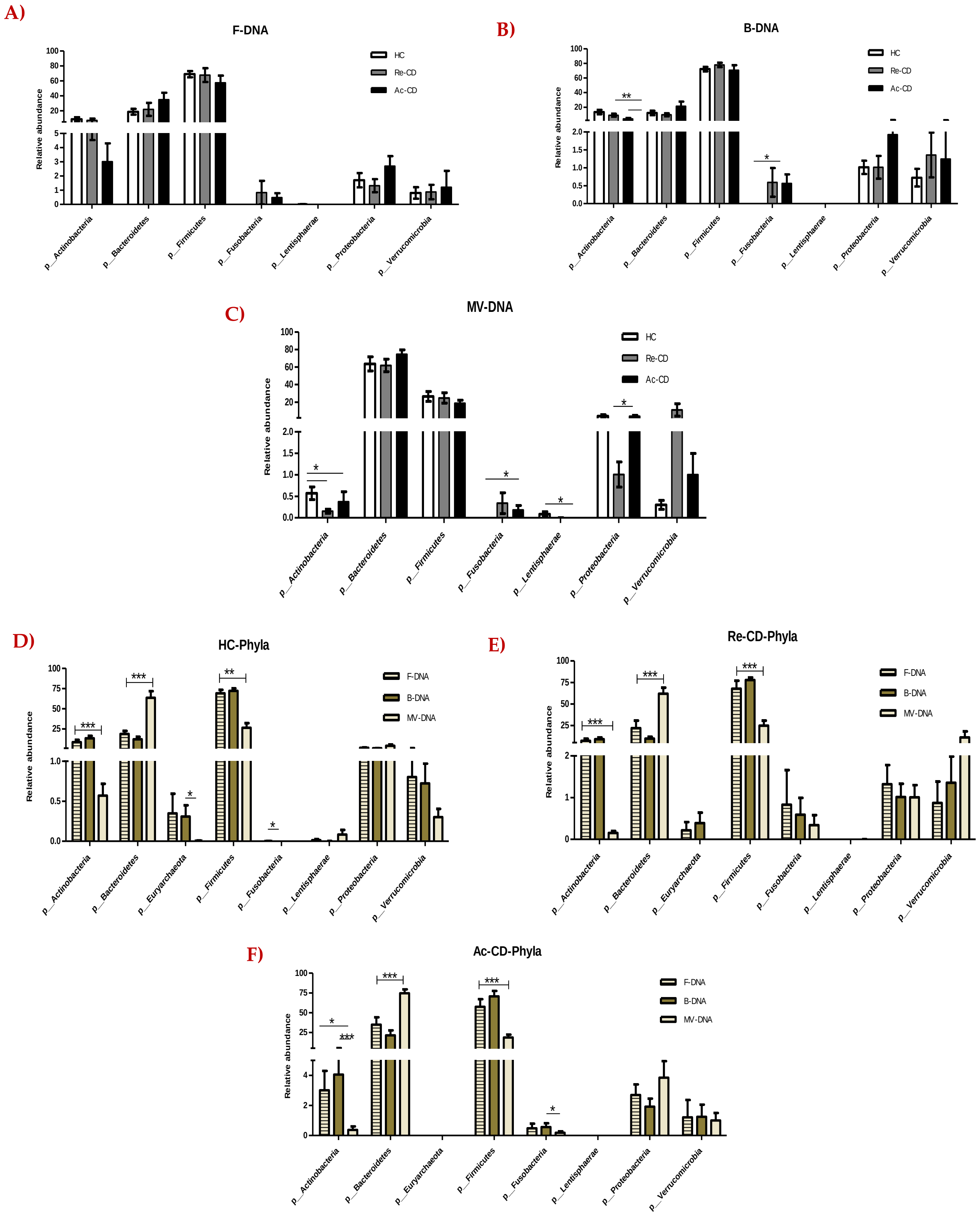

Figure S2: Comparison of relative abundances of the most dominants phyla. The graphs represent the relative differences between healthy controls and CD patients (remissive and active) based on DNA obtained A) from feces, B) from bacteria pellet, and C) from MVs. Firmicutes and Bacteroidetes which are the most abundant don't show significant differences, while Actinobactria , Fusobacteria, and Proteobacteria display significant changes especially in MV-DNA. The graphs D), E), and F) demonstrate altering in ratio between bacteria and their corresponding vesicles in HC, Re-CD, and Ac-CD, respectively. Results represent as means ±SEM. Student t-test is used for statistics; p-value indicates as (*<0.05, **<0.01, ***<0.001).

A)

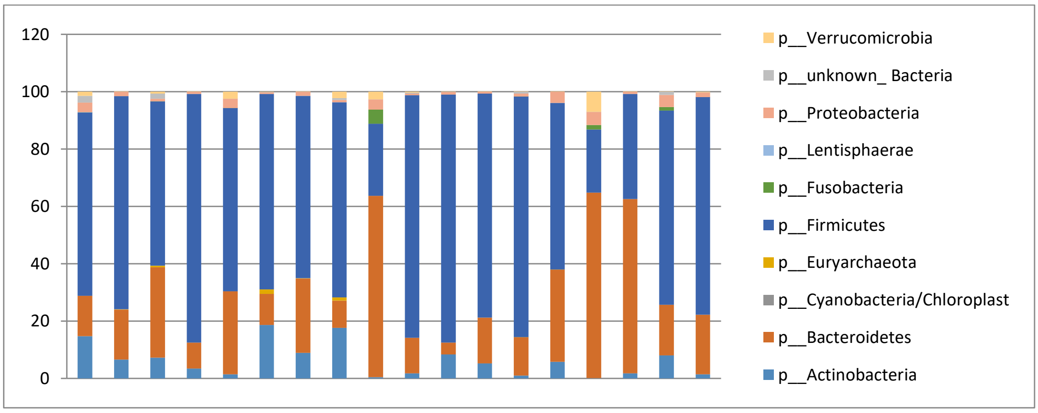

B)

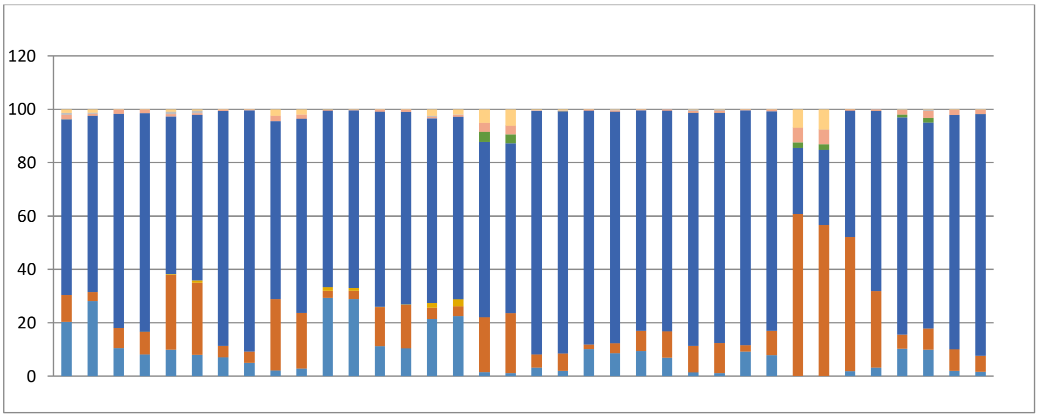

C)

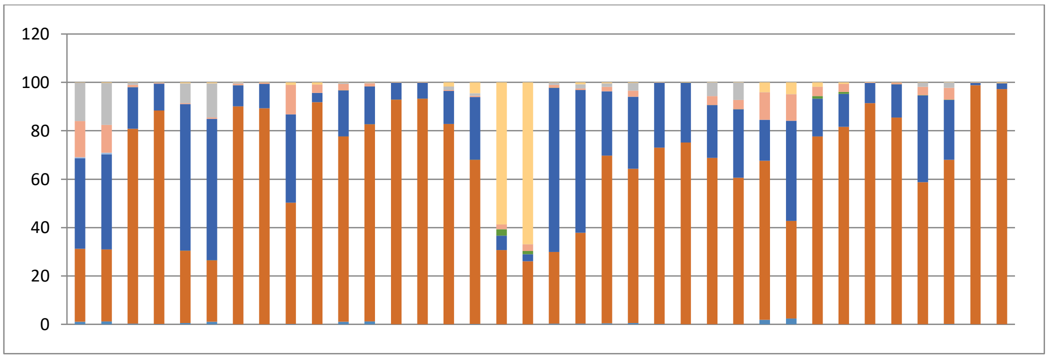

Figure S3: The microbial compositions of DNA at Phyla level. A) microbial composition of DNA obtained directly from fecal samples. bars 1-6 from healthy individuals, 7-12 from Crohn's patients in remissive state Re-CD, 13-18 from Crohn's patients in active state Ac-CD. B) microbial compositions of DNA obtained from bacterial pellet, each sample were analyzed twice (two different isolation from each individual fecal sample for reproducibility); bars 1-12 represents healthy individuals, 13-24 from Re-CD, and 25-36 from Ac-CD. C) Microbial composition of DNA isolated from MVs, all samples also analyzed twice, bars 1-12 from HC, 13-24 from Re-CD, and 25-36 from Ac-CD. Firmicutes phylum are more dominant in fecal and bacterial DNA while Bacteroidetes are abundant in MVs-DNA.

A)

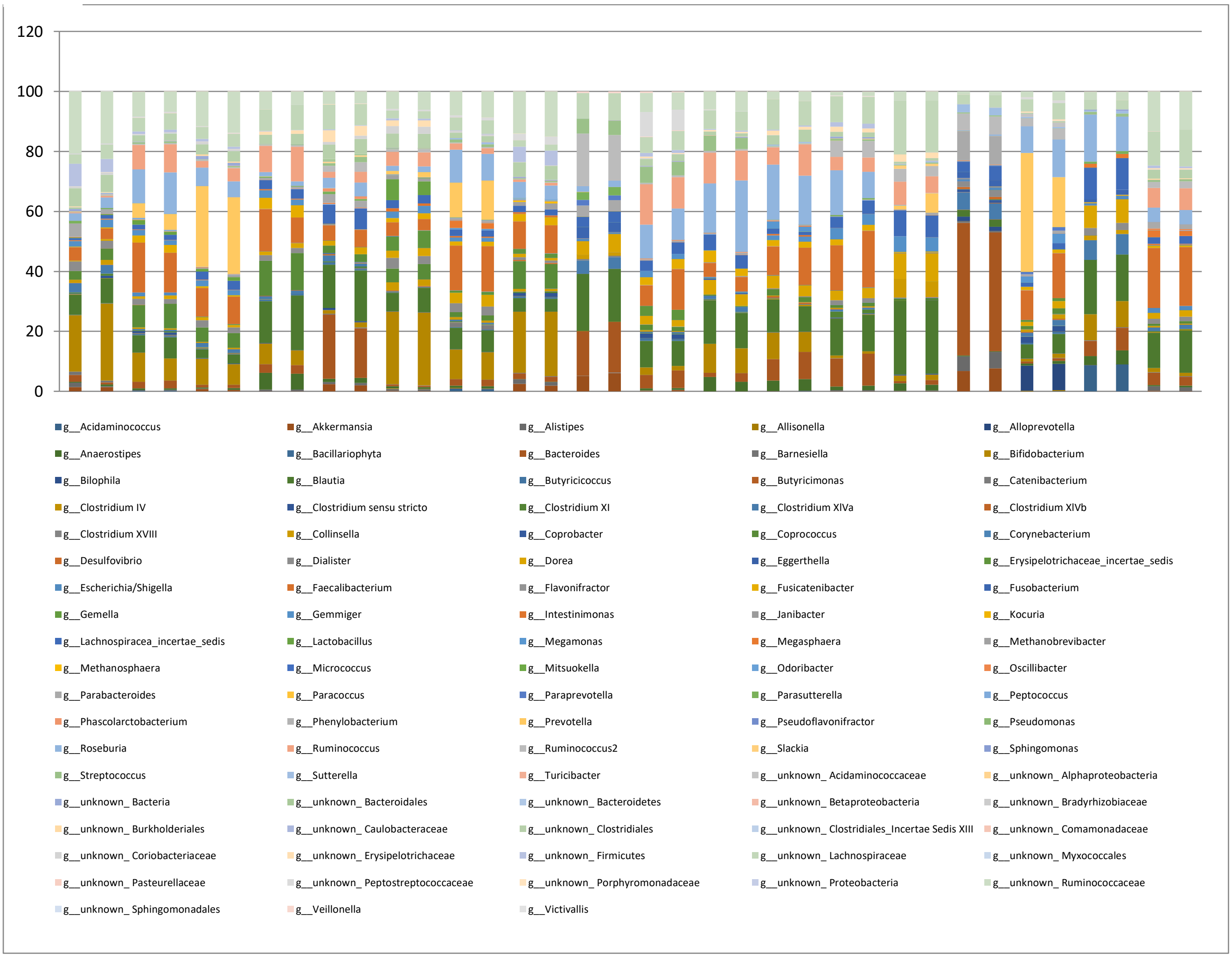

B)

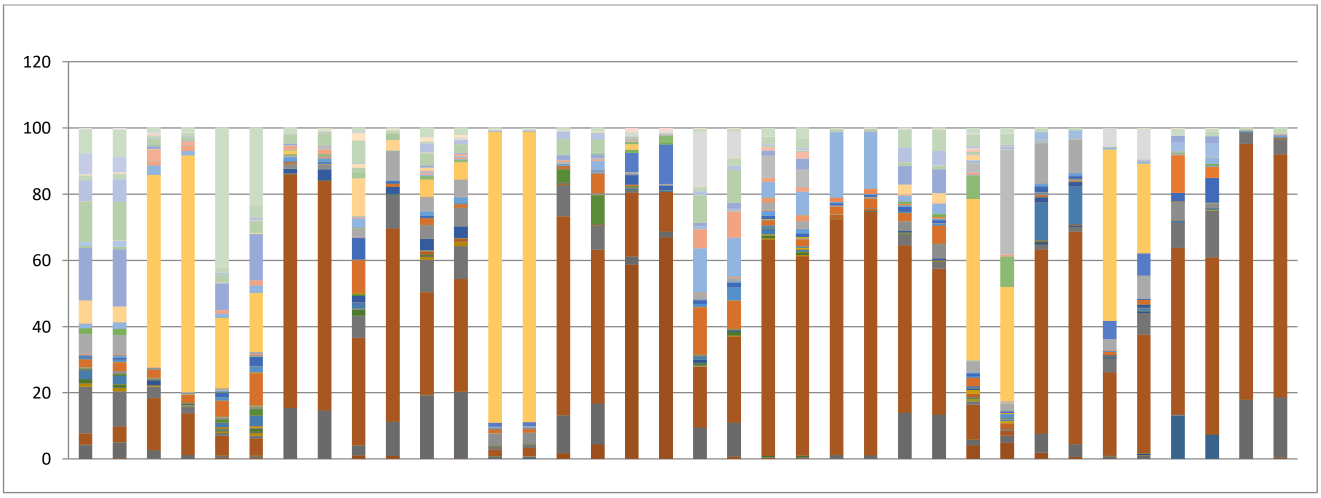

Figure S4: The relative abundance of microbial compositions at genus level. all samples also ran twice (two different isolation from each individual fecal sample for reproducibility). A) microbial compositions of DNA obtained from bacterial pellet; each sample were analyzed in twice. Bars 1-12 represents healthy individuals, 13-24 from Re-CD, and 25-36 from Ac-CD. B) Microbial composition of DNA isolated from MVs; bars 1-12 from HC, 13-24 from Re-CD, and 25-36 from Ac-CD. Each two bars in a row represent one individual.

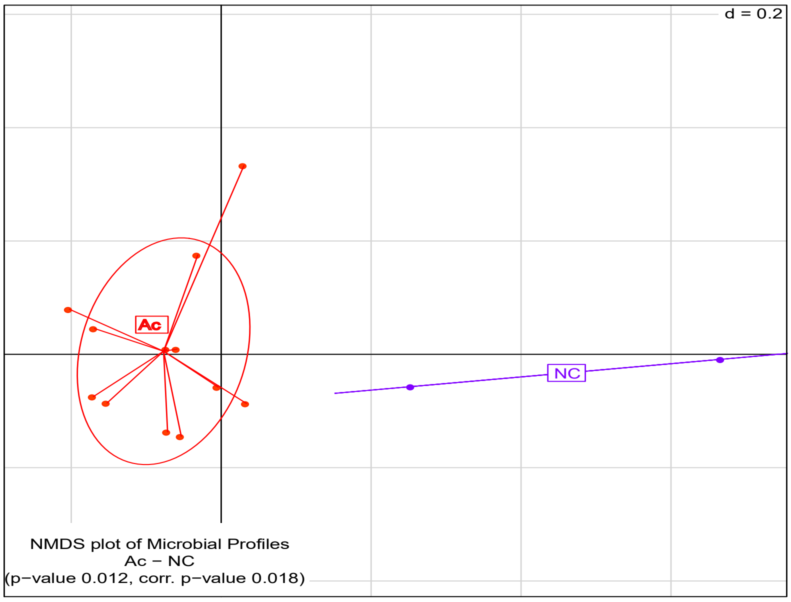

Figure S5: Beta diversity of highest negative controls compare to the lowest sequences reads of samples. It shows that the microbial compositions of negative controls have completely different compositions compare to the samples and the microbial compositions of samples are not driven by potential contaminated DNA.
